# Attenuating Diabetic Vascular and Neuronal Defects by Targeting P2rx7

**DOI:** 10.3390/ijms20092101

**Published:** 2019-04-29

**Authors:** Sofia Pavlou, Josy Augustine, Rónán Cunning, Kevin Harkin, Alan W. Stitt, Heping Xu, Mei Chen

**Affiliations:** Centre for Experimental Medicine, School of Medicine, Dentistry & Biomedical Science, Queen’s University Belfast, Belfast BT9 7BL, Northern Ireland, UK; s.pavlou@qub.ac.uk (S.P.); j.augustine@qub.ac.uk (J.A.); r.cunning@qub.ac.uk (R.C.); kharkin05@qub.ac.uk (K.H.); a.stitt@qub.ac.uk (A.W.S.); heping.xu@qub.ac.uk (H.X.)

**Keywords:** diabetic retinopathy, P2rx7, 3TC, retinal function

## Abstract

Retinal vascular and neuronal degeneration are established pathological features of diabetic retinopathy. Data suggest that defects in the neuroglial network precede the clinically recognisable vascular lesions in the retina. Therefore, new treatments that target early-onset neurodegeneration would be expected to have great value in preventing the early stages of diabetic retinopathy. Here, we show that the nucleoside reverse transcriptase inhibitor lamivudine (3TC), a newly discovered P2rx7 inhibitor, can attenuate progression of both neuronal and vascular pathology in diabetic retinopathy. We found that the expression of P2rx7 was increased in the murine retina as early as one month following diabetes induction. Compared to non-diabetic controls, diabetic mice treated with 3TC were protected against the formation of acellular capillaries in the retina. This occurred concomitantly with a maintenance in neuroglial function, as shown by improved a- and b-wave amplitude, as well as oscillatory potentials. An improvement in the number of GABAergic amacrine cells and the synaptophysin-positive area was also observed in the inner retina of 3TC-treated diabetic mice. Our data suggest that 3TC has therapeutic potential since it can target both neuronal and vascular defects caused by diabetes.

## 1. Introduction

Diabetic retinopathy (DR) is the most common complication of diabetes mellitus and accounts for 15–17% of total blindness in Europe and the United States [1,2]. DR consists of vascular and neuronal degeneration and it is believed that these two pathologies are closely linked. Beyond the vascular lesions that become apparent in the retinal fundus as diabetes progresses, it is now established that patients also experience reduced contrast sensitivity and impaired colour vision, which may precede the overt, clinically detectable vessel pathology [3,4,5,6]. Although these defects can all be linked to loss of integrity and cell–cell communication within the so-called neurovascular unit (NVU) [7], the precise nature of the pathogenic pathways involved needs to be fully elucidated. It has been speculated that neuronal damage, in itself, may cause oxidative stress and inflammation, which drive retinal microvascular degeneration [8,9], but this requires further investigation.

Purinergic receptor P2X, ligand-gated ion channel 7 (P2rx7) is a member of the purinergic P2X receptor family [10,11] and is expressed in many cell types [11,12]. Within the eyes, P2rx7 has been reported to be specifically expressed in retinal neurons under normal physiological conditions [13], although precisely how this receptor system is regulated in the retina during diabetes remains unknown [14]. Diabetes-related hyperglycaemia and hyperlipidaemia have been shown to upregulate P2rx7 expression or activity in skin fibroblasts in patients [15], while this receptor is also increased in the pancreatic α-cells [16], macrophages [16], kidneys [17], and corneas [18] of mice with streptozotocin (STZ)-induced diabetes. Diabetes-induced renal inflammatory pathology has been shown to be attenuated in mice lacking P2rx7 [19]. In vitro systems mimicking diabetes through high glucose exposure have shown that the P2rx7 pathway is closely associated with ATP-induced cell death in human primary fibroblasts [20]. Despite accruing evidence suggesting a possible role for P2rx7 in diabetes-related cell pathology, this pathway has not been extensively studied in DR. This study demonstrates that P2rx7 expression is upregulated in diabetic mouse retina and that a recently described P2rx7 inhibitor, called lamivudine (3TC), can protect against key aspects of retinal neuronal and vascular degeneration typical of DR.

## 2. Results

### 2.1. P2rx7 is Upregulated in Diabetic Mice

**Figure 1:**
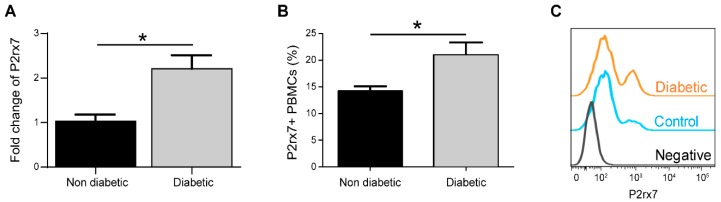
P2rx7 expression is upregulated in diabetic animals. Three-month old mice were rendered diabetic by streptozotocin (STZ) injections. One month after the onset of diabetes, blood was collected for flow cytometry and retinas were dissected and processed for RNA extraction and qRT-PCR. (**A**) Relative P2rx7 expression in non-diabetic and diabetic retinas. (**B**) Average percentage of P2rx7^+^ peripheral blood mononuclear cells (PBMC). (**C**) Representative histogram showing P2rx7^+^ cells. Student’s *t* test, * *p* < 0.05, *n* = 4–8.

The retinal expression of P2rx7 was assessed by qRT-PCR in control and diabetic animals. As shown in Figure 1A, there was a significant increase in P2rx7 expression one month after the onset of diabetes when compared to control animals. In addition, the percentage of P2rx7-positive peripheral blood mononuclear cells (PBMC) also increased in diabetic mice compared to controls (Figure 1B,C).

### 2.2. The Effect of 3TC on Diabetes and Circulating Immune Cells

**Figure 2:**
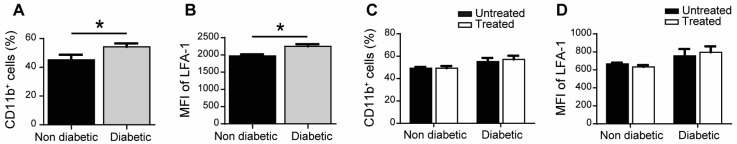
3TC treatment does not affect the circulating immune cells. Three-month old mice were rendered diabetic by STZ injections. One month after the onset of diabetes, blood was collected for flow cytometry (**A**,**B**). Mice were then administered 3TC daily by gavage feeding (185 mg/kg of body weight) and blood was collected for flow cytometry three months after treatment onset (**C**,**D**). (A) Average percentage of CD11b^+^ cells and (**B**) mean fluorescent intensity (MFI) for LFA-1, one month after the onset of diabetes. (**C**) Average percentage of CD11b^+^ cells and (**D**) MFI for LFA-1, four months after the onset of diabetes, with or without 3TC administration. (**A**,**B**) Student *t* test, * *p* < 0.05; (C,D) Two-way ANOVA, *n* = 6.

To further understand the role of P2rx7 in the pathogenesis of DR, 3TC was administered daily, one month after the onset of diabetes for five months. Blood glucose levels, circulating immune cells, and retinal pathologies were then examined. 3TC treatment did not affect blood glucose (>18 mmol/L for both treated and untreated conditions) or glycated haemoglobin Hba1c (untreated diabetic: 115.4 ± 2.93 mmol/mol; treated diabetic: 100 ± 10.69 mmol/mol). However, diabetes led to an increase in the proportion of circulating CD11b^+^ cells and an upregulation of the adhesion molecule LFA-1 on CD11b^+^ cells (Figure 2A,B). Three-month 3TC treatment did not affect the proportions of circulating immune cells, including CD11b^+^ myeloid cells (Figure 2C), CD3^+^ T cells, B220^+^ B cells, and CD56^+^ NK cells (data not shown) in control and diabetic mice. The treatment also did not affect the expression of adhesion molecules, such as LFA-1 (Figure 2D).

### 2.3. The Effect of 3TC on Visual Function

**Figure 3:**
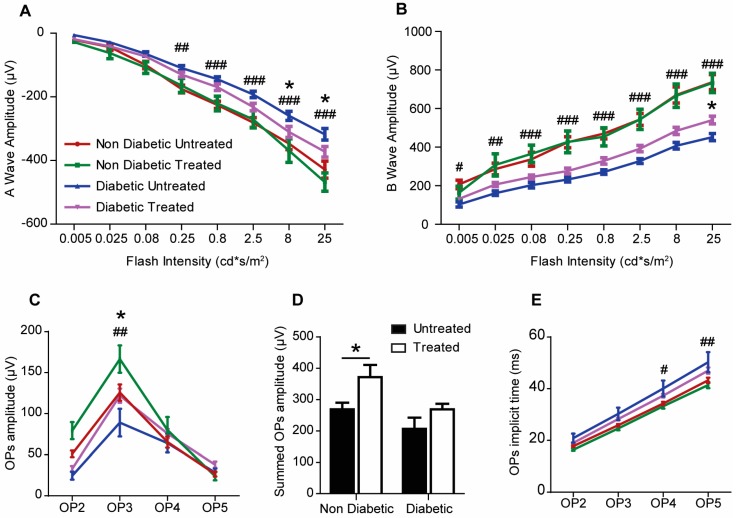
The deterioration of retinal function is attenuated by 3TC treatment in diabetic mice. Three-month old mice were rendered diabetic by STZ-injections. One month after the onset of diabetes, mice were administered 3TC daily by gavage feeding for five months (185 mg/kg of body weight). Electroretinography (ERG) was performed at the end of the study. (**A**) Average a-wave amplitude at different flash intensities. (**B**) Average b-wave amplitude at different flash intensities. (**C**) Average amplitude of each oscillatory potential (OP). (**D**) Average summed OP amplitudes. (**E**) Average implicit time for each of the OP. Data are shown as mean ± SEM. *n* = 6–16 eyes per experimental condition. Two-way ANOVA with Tukey’s multiple comparisons test for A–C and E. Two-way ANOVA with Sidak’s test for D. # *p* < 0.05, ## *p* < 0.01, and ### *p* < 0.001 between non-diabetic untreated and diabetic untreated animals; * *p* < 0.05, ** *p* < 0.01, and *** *p* < 0.001 between diabetic untreated and diabetic treated animals.

The a- and b-waves of electroretinography (ERG) were not affected by 3TC treatment in non-diabetic mice (Figure 3A,B). However, this treatment significantly increased the OP (oscillatory potential) amplitudes in non-diabetic mice (Figure 3C,D). Diabetic mice had impaired a- and b-waves amplitudes, reduced OP3 amplitude and prolonged OP implicit time (Figure 3A–E). 3TC treatment significantly improved a-wave (Figure 3A), b-wave (Figure 3B), and OP3 amplitude (Figure 3C), suggesting that P2rx7 inhibition improved visual function in diabetic mice.

### 2.4. The Effect of 3TC on Diabetes-Induced Retinal Neurodegeneration

**Figure 4:**
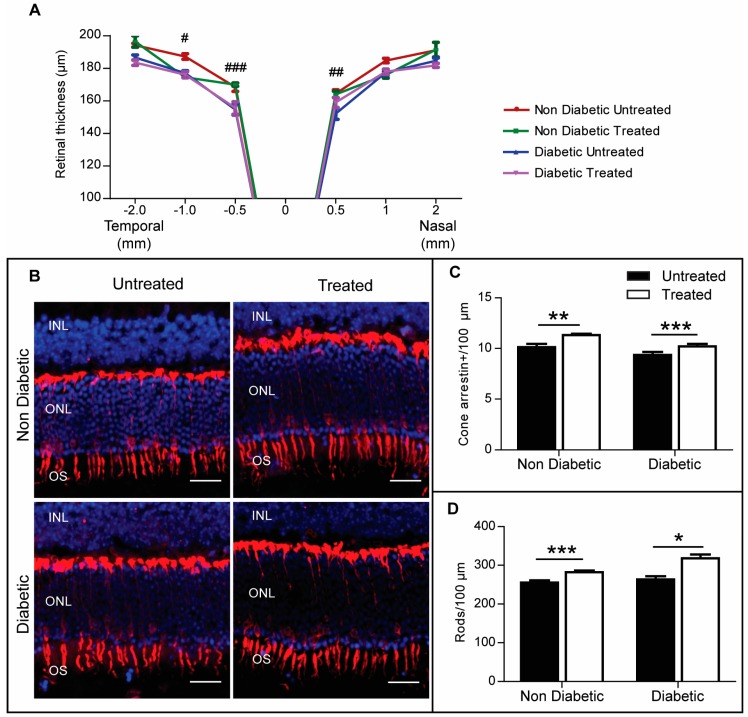
3TC protects photoreceptors from age-related degeneration. (**A**) Average retinal thickness across the temporal-nasal axis acquired via quantitative spectral domain optical coherence tomography (SD-OCT), *n* = 6 eyes per experimental condition. Two-way ANOVA with Tukey’s multiple comparisons test. # *p* < 0.05, ## *p* < 0.01, and ### *p* < 0.001 between non-diabetic untreated and diabetic untreated animals. (**B**) Cryosections from each experimental condition were stained for cone arrestin (red) and counterstained with DAPI (blue). The average number of cones (cone arrestin-positive cells) or rods (DAPI-positive cells in the ONL minus cones) were quantified and normalised to 100 μm of retina length. INL: inner nuclear layer; ONL: outer nuclear layer; OS: outer segment. Scale bar: 25 μm. (**C**) Average number of rods per 100 μm. (**D**) Average number of cones. Data are shown as mean ± SEM. *n* > 40 images from 3–5 different animals per experimental condition. Two-way ANOVA with Sidak’s test, * *p* < 0.05, ** *p* < 0.01, and *** *p* < 0.001.

Using quantitative spectral domain optical coherence tomography (SD-OCT) to evaluate the retinas of animals in the experimental groups, it was apparent that the thickness of the neuronal retina was reduced in diabetic mice compared to that in control mice. 3TC treatment did not affect the retinal thickness in control or diabetic mice (Figure 4A).

With ERG data suggesting that P2rx7 inhibition was protective against diabetes-induced retinal neuronal damage, further post-mortem analysis was conducted to understand which neuronal populations were protected by 3TC. In particular, since the ERG a-wave reflects the function of photoreceptors and this parameter was shown to be protected by P2rx7 inhibition in the diabetic group, the number of arrestin-positive cone cells was analysed. There was no significant difference in the number of cone cells in diabetic retina compared with non-diabetic controls (Figure 4B,C). Interestingly, 3TC treatment significantly maintained the number of cone cells in both diabetic and non-diabetic animals (Figure 4B,C). A similar effect was observed in rod cells (Figure 4D), suggesting that P2rx7 inhibition could play an important role in protecting photoreceptors from age-induced degeneration.

**Figure 5:**
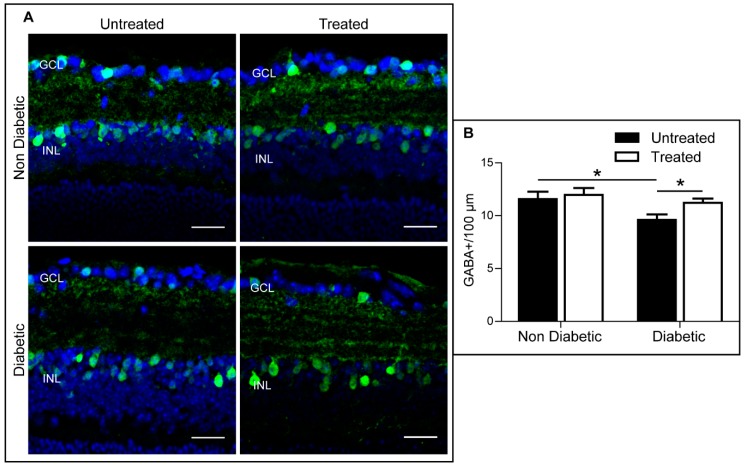
3TC attenuates diabetes-mediated GABAergic amacrine cell loss. Cryosections from each experimental condition were stained for GABA (green) and counterstained with DAPI (blue). The average number of GABAergic amacrine cells within the INL were quantified and normalised to 100 μm of retina length. (**A**) Representative images for each condition. GCL: ganglion cell layer; INL: inner nuclear layer. Scale bar: 25 μm. (**B**) Average number of GABAergic amacrine cells per 100 μm. Data are shown as mean ± SEM. *n* > 30 images from 3–5 different animals per experimental condition. Two-way ANOVA with Sidak’s test, * *p* < 0.05.

**Figure 6:**
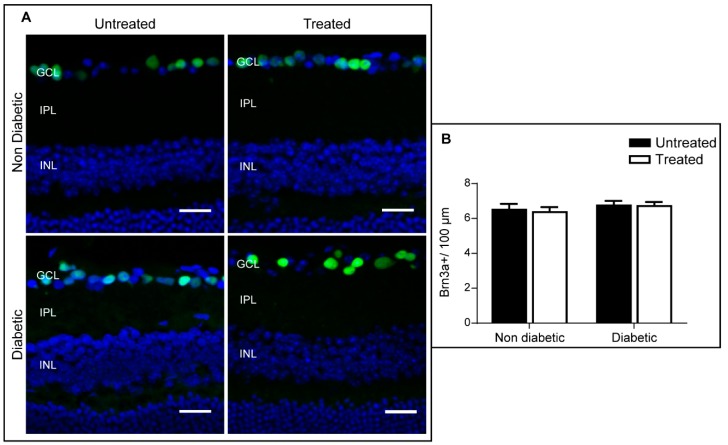
Retinal ganglion cell number is unaffected by STZ-mediated diabetes. Cryosections from each experimental condition were stained for Brn3a (green) and counterstained with DAPI (blue). The average number of Brn3a^+^ ganglion cells were quantified and normalised to 100 μm of retina length. (**A**) Representative images for each condition. GCL: ganglion cell layer; IPL: inner plexiform layer; INL: inner nuclear layer. Scale bar: 25 μm. (**B**) Average number of Brn3a^+^ cells per 100 μm. Data are shown as mean ± SEM. *n* > 30 images from 3–5 different animals per experimental condition. Two-way ANOVA with Sidak’s test.

The number of GABAergic amacrine cells was significantly reduced in diabetic retina when compared to non-diabetic retina (Figure 5), and this defect was attenuated in diabetic animals that had received 3TC treatment (Figure 5). 3TC treatment did not affect the number of GABA-positive cells in non-diabetic animals (Figure 5). No significant difference was observed in the number of ganglion cells (Brn3a-positive) in any of the four groups (Figure 6).

### 2.5. The Effect of 3TC on Retinal Synaptophysin Expression in Control and Diabetic Mice

**Figure 7:**
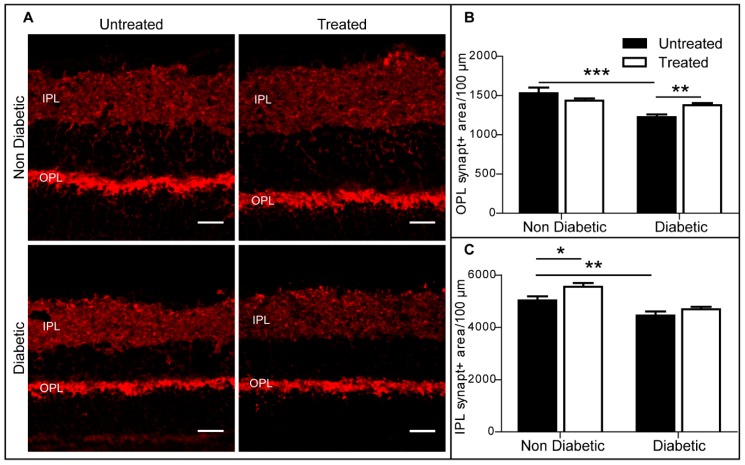
Synaptophysin expression was protected in diabetic retina treated with 3TC. (**A**) Representative images showing synaptophysin (red) in OPL and IPL in different groups. OPL: outer plexiform layer; IPL: inner plexiform layer. Scale bar: 25 μm. (**B**) Synaptophysin-positive area in OPL was reduced in diabetic retina, and 3TC can protect the degeneration of synaptophysin. (**C**) The expression of synaptophysin-positive area was reduced in diabetic retina, although 3TC did not show significant protection. Data are shown as mean ± SEM. *n* > 24 images from 3–5 different animals per experimental condition. Two-way ANOVA with Sidak’s test, * *p* < 0.05, ** *p* < 0.01, and *** *p* < 0.001.

Synaptophysin is ubiquitously expressed at the synapses. Within the retina, synaptophysin is detected in both outer plexiform layer (OPL) and inter plexiform layer (IPL). We quantified the synaptophysin-positive area within the OPL or IPL and normalised it to 100 μm of retinal length. In the OPL, there was a significant reduction in synaptophysin-positive area in diabetic mice compared to non-diabetic mice (Figure 7A,B), and this reduction was significantly attenuated by 3TC treatment (Figure 7A,B). The expression of synaptophysin in the IPL was also reduced in diabetic retina compared to non-diabetic retina. 3TC treatment increased IPL synaptophysin in control non-diabetic mice, but not diabetic mice (Figure 7A,C).

### 2.6. The Effect of 3TC on Diabetes-Induced Acellular Capillaries

**Figure 8:**
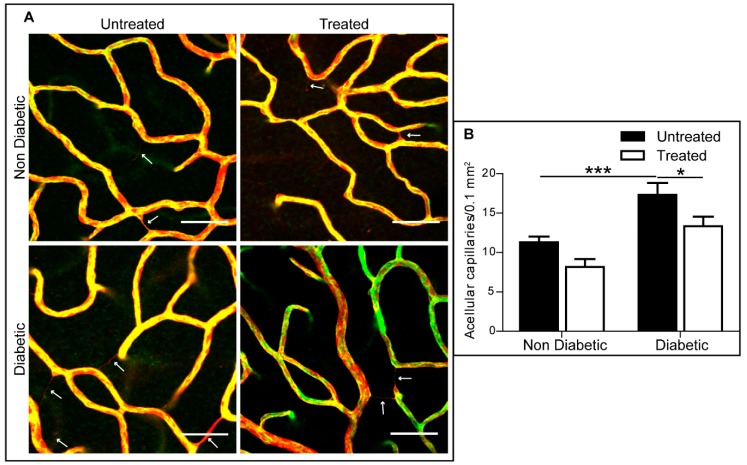
3TC prevented acellular capillary development in diabetic mice. (**A**) Representative images showing acellular capillaries in diabetic retina and non-diabetic control with or without 3TC treatment. Arrows demonstrating acellular capillaries, which were isolectin B4 (green)-negative and collagen IV (red)-positive. Scale bar: 50 μm. (**B**) Quantification of acellular capillaries in different groups. Data are shown as mean ± SEM. *n* > 13 images from 2–3 different animals per experimental condition. Two-way ANOVA with Sidak’s test, * *p* < 0.05, and *** *p* < 0.001.

Acellular capillaries are an early sign of diabetes-induced retinal vasculopathy, and this was confirmed in the diabetic animals after six months of hyperglycaemia (Figure 8). P2rx7 inhibition using 3TC significantly prevented acellular capillary formation (Figure 8).

## 3. Discussion

Previous studies have shown that P2rx7 is expressed in retinal neurons, including ganglion and amacrine cells, but not glial cells [13,21,22]. In a defined disease context, this receptor can mediate pro-apoptotic signalling, formation of large plasma membrane pores, and loss of cellular integrity [23,24]. Diabetes has been shown to upregulate P2rx7 expression in various cells [15,25], and in the current study, we demonstrated that expression of this receptor was increased in the diabetic retina. Lamivudine (3TC), a nucleoside reverse transcriptase inhibitor with well-described P2rx7 inhibition properties [26,27,28], reduced diabetes-induced retinal neuronal and vascular degeneration. Our results suggest that the P2rx7 receptor pathway plays a significant role in the pathogenesis of diabetic retinopathy.

Retinal neuronal degeneration, including ganglion cell death and photoreceptor degeneration, has been detected at the early stages of diabetes, although the underlying mechanism remains elusive. Excessive ATP has been detected in the vitreous of patients with DR [29]. ATPs, released by the diseased retina, can directly activate the P2rx7 receptors on neurons and induce cell death. Intraocular injection of ATP led to photoreceptor death and retinal degeneration [30,31]. Since 3TC can inhibit P2rx7 activity [26,27,28], and is widely used to treat HIV patients and proven to be safe, we treated diabetic mice with 3TC. The treatment affected neither the blood glucose/HbA1c levels nor the circulating immune cells, but significantly reduced retinal neuronal and vascular degeneration. The results suggest that 3TC may protect retinal cells by targeting the P2rx7 receptor pathway. Inflammation is known to play an important role in the pathogenesis of DR [32,33,34], and recent studies have shown that the nucleoside reverse transcriptase inhibitors are able to suppress inflammation in a P2rx7-dependent manner [26,27]. Further studies are needed to understand whether 3TC protected diabetic retina through suppressing inflammation.

A significant reduction in the number of GABAergic amacrine cells and the area of synaptophysin were observed in diabetic mice when compared to non-diabetic controls. Diabetes-induced ganglion cell death is widely reported in the literature [35,36,37]. A decrease in synaptophysin at the protein level was reported previously in diabetic mice [38]. Synaptophysin is a synaptic vesicle protein important for visual function [38]. Interestingly, 3TC treatment significantly reduced diabetes-induced amacrine death and synaptophysin reduction. The results suggest that P2rx7 receptor pathway may have distinct roles in different neurons under diabetic conditions.

In our study, we found that 3TC treatment significantly prevented the decline in the number of photoreceptors in normal non-diabetic mice. A previous study by Kolesnikov et al. [39] has reported an aged-dependent deterioration of rods in 29-month-old mice when comparing with 4-month-old mice. P2rx7-mediated photoreceptor death has been observed in vitro and in vivo (i.e., followed intraocular injections of BzATP in mice) [31]. Our results suggest that 3TC can protect photoreceptors from age-related degeneration.

Acellular capillary formation is a hallmark of diabetes-induced retinal vasculopathy in mice, and it was found that 3TC treatment significantly decreased the occurrence of this important lesion in diabetic mice. Sugiyama et al. [25] demonstrated that retinal capillaries from diabetic rats were more susceptible to P2rx7-mediated cell death than those from non-diabetic controls, and it was reported that this was due to diabetes increasing the sensitivity, rather than the number, of P2rx7 receptors [25]. Our data suggest that inhibiting the activity of P2rx7 receptor by 3TC can protect endothelial cells for diabetes-mediated cell death.

Current managements of DR focus exclusively on end-stage complications. Since 3TC is widely used in the clinic for the treatment of HIV and hepatitis and proven to be safe, it is worth further investigating the preventive or therapeutic potential of 3TC in DR management in clinical settings.

## 4. Methods

### 4.1. Animals

Male C57BL/6J mice were bred and maintained in the Biological Service Unit at Queen’s University Belfast in a 12 h light/12 h dark cycle with free access to water and chows. The use of animals followed the UK Home Office Animal (Scientific Procedures) Act 1986 and was approved by the local Ethical Review Committee (Home Office Project Licence 2773, approved in October 2014).

### 4.2. STZ-Mediated Type 1 Diabetes

Diabetes was induced in 12-week-old male C57BL/6J mice (~25 g) by five daily intraperitoneal injections of 50 mg/kg streptozotocin (STZ) in freshly prepared 0.1 M citrate buffer (pH 4.5). Control animals received citrate buffer alone. One week after injections, glucose level was determined using FreeStyle Lite Blood Glucose Test strips (Abbott, Oxfordshire, UK). Blood glucose levels and weights were monitored biweekly throughout the study. Glycated haemoglobin (Hba1c) levels were assessed at the end of the experiment using the A1CNow^+^ system (PTS Diagnostics, Indianapolis, IN, USA), according to manufacturer’s instructions.

### 4.3. 3TC Administration

One month after the onset of diabetes (16 weeks of age), control and diabetic mice were treated with lamivudine, also known as 3TC, daily for 5 months (185 mg/kg of body weight) [26]. 3TC was dissolved in water and administered orally.

### 4.4. Electroretinography

Scotopic electroretinography (ERG) responses were evaluated in treated and untreated, control and diabetic mice at 9 months of age. Mice were dark-adapted overnight, and all procedures were performed under dim red light (<1 lx). Pupils were dilated using 1% atropine and 2.5% phenylephrine (Chauvin, Essex, UK). Mice were anesthetised via intraperitoneal injection of ketamine hydrochloride (60 mg/kg, Vetoquinol UK Ltd, Northamptonshire, UK) and xylazine hydrochloride (5 mg/kg, Bayer HealthCare, KVP Pharma + Veterinar Produjte GmbH, Kiel, Germany). ERG was performed using an Espion ERG Diagnosys machine (Diagnosys LLC, Littleton, MA, USA), according to manufacturer’s instructions. Briefly, the mouse was placed on top of a heating pad within the Ganzfeld bowl illuminator and monitored using an infrared camera. ERG responses were recorded using corneal electrodes placed on each eye, a forehead reference electrode and a tail ground electrode. Eight light intensities ranking from 0.008 to 25 cd·sec/m^2^ were used. The average of 4 responses for each light intensity is shown. Oscillatory potentials (OPs) were recorded using 25 cd·sec/m^2^ light intensity and calculated based on the manufacturer’s calibrations. Between 6 and 16 eyes were analysed for each experimental condition.

### 4.5. Spectral Domain Optical Coherence Tomography (SD-OCT)

Pupils were dilated, and mice were anesthetised as described above. SD-OCT was conducted using the Spectralis Heidelberg OCT system (Heidelberg Engineering, Heidelberg, Germany), according to manufacturers’ instructions. At least 6 eyes per group were analysed.

### 4.6. Flow Cytometry

Peripheral blood was collected and processed for flow cytometry as previously described [40]. The cell surface markers LFA-1 (clone 2D7) and CD11b (clone M1/70) were evaluated. Data were collected using the BD FACSCanto II (BD Biosciences, Berkshire, UK) and analysed using the FlowJo software (Tree Star, Inc. Ashland, OR, USA). Blood samples from 6 mice per group were analysed.

### 4.7. Immunofluorescence Staining

Eyes were fixed in 2% paraformaldehyde for 2 h at room temperature and 16 μm-thick cryosections were washed and incubated in primary antibodies (Table 1) overnight at 4 °C. After several washes, sections were then incubated in secondary antibodies (Table 1) for 1 h at room temperature and mounted using VECTASHIELD supplemented with DAPI (Vector Labs, Burlingame, CA). Stained sections were examined by C1 Nikon confocal microscopy (Nikon UK Ltd., Surrey, UK).

Eyes were fixed for 20 min in 2% paraformaldehyde at room temperature. Retinas were then dissected and stored in ice-cold methanol in −20 °C until used. For staining, retinas were washed extensively with PBS, permeabilised for 3 h using 1% Triton X-100 in PBS, and incubated with primary antibodies in 1% BSA and 0.1% Triton X-100 in PBS overnight at 4 °C (Table 1). They were then washed using 0.2% Triton X-100 in PBS, incubated with secondary antibodies (Table 1) for 3 h at room temperature, washed and mounted using VECTASHIELD (Vector Laboratories, Burlingame, CA, USA).

### 4.8. Real Time qRT-PCR

RNA was extracted from retinas 1 month after the onset of diabetes using RNeasy mini kit (Qiagen, Manchester, UK). cDNA libraries were generated using Superscript II Reverse transcriptase kit (Thermo Fisher Scientific, Waltham, MA, USA). qRT-PCR was performed using LightCycler 480 II (Roche-Diagnostic, Basel, Switzerland). Validated TaqMan probes were purchased from Roche (Basel, Switzerland): P2rx7 (315374), 18S (307906). RNA from 4 non-diabetic control and 8 diabetic animals was analysed.

### 4.9. Retinal Cell Quantifications

Fluorescent images were acquired with constant settings for each antibody. At least 5 images with 10 optical steps (1 μm each step) per section from the central region of the retina were acquired. Eyes from 3 to 5 mice were processed for each study group. The data are presented as average ± standard error of the mean (SEM), normalised to 100 μm of retinal length.

Cone arrestin was used to quantify the number of cone cells. The number of rod cells was calculated by subtracting the number of cone cells from the total DAPI-positive cells in the outer nuclear layer. GABA-positive cells in the INL used to quantify GABAergic amacrine cells. Brn3a-positive cells in the ganglion cell layer (GCL) represent ganglion cells. Synaptophysin was used to study the synapses and the area of synaptophysin-positive staining in the OPL, and IPL was measured and normalised to 100 μm of retinal length. 

### 4.10. Statistical Analysis

Statistical analysis was performed using GraphPad Prism version 6 for Windows (GraphPad Software, San Diego, CA, USA). Student’s *t* tests were performed for analysis and comparisons between two groups. Two-way analysis of variance (ANOVA), followed by Tukey’s (for repeated measurements—RM) or Sidak’s (for independent measurements) multiple comparison test was used to investigate the effect of diabetes with or without treatment. Data are represented as mean ± SEM and *p* < 0.05 was considered statistically significant.

## Figures and Tables

**Table 1:** Primary Antibodies and Secondary Antibodies.

Antibody	Dilution	Source	Retinal Localisation
Cone arrestin	1/1000	Merck Millipore	Cone photoreceptors
GABA	1/500	Sigma	GABAergic amacrine cells
Brn3a	1/50	SantaCruz	Ganglion cells
Synaptophysin	1/200	Abcam	Presynaptic elements
Isolectin B4	1/50	Vector Labs	Endothelial cells
Collagen IV	1/75	Bio Rad	Basal lamina in blood vessels
Donkey anti-rabbit Alexa Fluor 488	1/200	Jackson ImmunoResearch	Secondary Antibody
Donkey anti-rabbit Alexa Fluor 594	1/200	Jackson ImmunoResearch	Secondary Antibody
Donkey anti-goat Alexa Fluor 546	1/200	Jackson ImmunoResearch	Secondary Antibody
Streptavidin Alexa Fluor 488	1/100	Life Technologies	Secondary Antibody

## Data Availability

The datasets generated and analysed during the current study are available from the corresponding author on reasonable request.
